# Analytical methods for assessing antimicrobial activity of nanomaterials in complex media: advances, challenges, and perspectives

**DOI:** 10.1186/s12951-023-01851-0

**Published:** 2023-03-20

**Authors:** Xuzhi Zhang, Xiangyi Hou, Liangyu Ma, Yaqi Shi, Dahai Zhang, Keming Qu

**Affiliations:** 1 Laboratory for Marine Fisheries Science and Food Production Processes, Pilot National Laboratory for Marine Science and Technology, Yellow Sea Fisheries Research Institute, Chinese Academy of Fishery Sciences, Qingdao, 266071 China; 2 School of Marine Ecology and Environment, Shanghai Ocean University, Shanghai, 201306 China; 3 Key Laboratory of Marine Chemistry Theory and Technology, Ministry of Education, Ocean University of China, Qingdao, 266100 China

**Keywords:** Nanomaterials, Antimicrobial activity, Complex matrices, Assessment, Analytical methods

## Abstract

Assessing the antimicrobial activity of engineered nanomaterials (ENMs), especially in realistic scenarios, is of great significance for both basic research and applications. Multiple analytical methods are available for analysis via off-line or on-line measurements. Real-world samples are often complex with inorganic and organic components, which complicates the measurements of microbial viability and/or metabolic activity. This article highlights the recent advances achieved in analytical methods including typical applications and specifics regarding their accuracy, cost, efficiency, and user-friendliness. Methodological drawbacks, technique gaps, and future perspectives are also discussed. This review aims to help researchers select suitable methods for gaining insight into antimicrobial activities of targeted ENMs in artificial and natural complex matrices.

## Introduction

The twenty-first century has witnessed rapid developments in nanotechnology. Applications of engineered nanomaterials (ENMs) continue to expand in construction, electronics, agriculture, environment, food, consumer product, health care, energy, and medicine [1–3]. These ENMs have great potential to improve quality of life. Their small size, high surface energy, high surface-to-volume ratio, and high grain boundary atomic rate make them dissimilar from their bulk phase and therefore favorable for use [2–4]. The same properties also lead to concerns about potential toxicological and adverse effects [4–7]. They are reportedly prone to interactions with cell membranes, proteins, DNA, and cellular organelles [2]. Will the societal and commercial advantages be outweighed by potential disadvantages? To answer this question, accurate insight into the eco-toxicity of ENMs is needed [6–9]. In recent decades, extensive research has investigated the toxicity of ENMs towards biota [7, 9] to provide reliable data for risk assessments [6, 10, 11].

Bacteria are useful bio-reporters for assessing the toxicity of ENMs [12, 13] because they are (1) very important for geobiochemistry and humans, (2) ubiquitous, (3) susceptible to changes of environmental factors, and (4) easy to handle. The simple structures and short life-cycles offer clear advantage over other bio-reporters for performing high throughput screening [12–14]. Moreover, many kinds of ENMs are designed as novel antibiotics to counteract bacteria [2, 3, 15–19].

The toxicity of ENMs towards bacteria has been studied extensively [13–16]. In most cases, the effects of ENMs on bacterial viability and/or metabolic activity are analyzed in simple laboratory media [2, 15, 20–24]. It is increasingly clear that their toxic efficiency depends on not only the shape, size and physicochemical properties of ENMs themselves and target bacterial cells but also on physical factors and chemical/biological components in the test scenarios [2, 11, 16, 25] such as temperature, pH, ionic strength, organic matters, and inorganic matter [25–29]. In most applications and concerned eco-systems, ENMs encounter bacterial cells in highly complex media with various inorganic and organic components [2]. The results of these interactions cannot be predicted with data obtained in simple laboratory media [30].

Samples from realistic scenarios, e.g., soil, natural water, sediment, sludge, consumer products, food, blood, and biotic tissue, are often complex in physical and chemical features; and thus complicate measurements of microbial viability and/or metabolic activity. Determining the antimicrobial activity of ENMs in realistic samples is far more difficult than in simple laboratory media. As has been well-reviewed by Westmeier et al. [2], though many classical and emerging methods can be used to determine the antimicrobial activity of ENMs, only a few of these methods can complete tasks where complex samples are involved [11, 16, 31]. Currently, comprehensive information on these determination methods is not available. In this review, we do not attempt to cover the substantial body of relevant literature in this field, but rather to review the recent advances achieved in analytical methods for assessing the antimicrobial activity of ENMs in complex matrices. The accuracy, cost, efficiency and user-friendliness of applied and emerging methods are described along with drawbacks, technique gaps and future perspectives. All figures are reprinted with permission from related publishers/authors.

## Applied methods

Methods used to assess the antimicrobial activity of ENMs usually refer to the antibiotic susceptibility testing (AST). In general, phenotypic AST methods provide a direct indication of the susceptibility of a given microbe to an agent at defined concentrations; in some cases, such methods provide a quantitative assessment of the minimal inhibitory concentration (MIC) of the antibiotic. In contrast, genotypic methods are used to describe methods that detect proteomic or genomic signatures that predict antimicrobial resistance [32]. To date, there are few reports on genotypic methods for assessing the antimicrobial activity of ENMs in complex matrices. Here, we divide these phenotypic methods into two patterns: off-line measurements and on-line measurements. For the former, microbial viability and/or metabolic activity are measured after the model cells are exposed to target ENMs for expected terms. In other words, information on the inhibition, if there is any, results from endpoint measurements of microbial viability and/or metabolic activity. For the latter, microbial viability and/or metabolic activity are measured on-line during the exposure process of ENMs to microbes without sampling operation. Information on the inhibition, if there is any, results from the microbial response in an undisturbed incubation. Note that the complexity of samples from realistic scenarios often makes analytical methods ill-suited for accurately assessing the antimicrobial activity of ENMs [31]. Moreover, the physicochemical properties of ENMs are significantly different from that of antibiotics. Therefore, each method has differences between the AST of common antibiotics and the antimicrobial activity test of ENMs especially when pretreatments are needed.

In real-world samples, the cell structure and physiological property of microbes vary widely. The physicochemical properties of present ENMs are also variable [30], and biotic and abiotic matter are complex and variable as well [16]. As such, researchers must select suitable approaches for these various conditions. To obtain optimal results, various pretreatment and optimization steps are needed. In some cases, a combination of different methods can compensate for the drawbacks and limitations of individual methods [2]. It is impossible to discuss analytical methods in detail for all types of ENMs and microbes. Here, we present typical off-line methods and on-line methods that are essential for assessing the antimicrobial activity of ENMs in complex matrices. The principles mentioned herein aim to help the reader to understand best practices in assessing the antimicrobial activity of ENMs.

Non-biased approaches are critical when discussing the performance of various analytical methods. This is not an easy task due to the diversity of the reported protocols, of the ENMs to be assessed, of the species of microbes to be evaluated, and of the realistic scenarios of interest. Here, important criteria for comparing different methods include both experimental feasibility and practical values.

### Off-line measurement

After short-term or long-term exposure to target ENMs, the viabilities and/or metabolic activities of microbes are measured at the endpoint with or without pretreatments. Media can be sampled for dozens of times during the exposure process for longitudinal information on microbial viability and/or metabolic activity. These time-dependent values are often used to understand how target ENMs affect the kinetics of microbial growth.

Endpoint measurements (i.e., bacterial viability) obtained after microbial exposure to target ENMs for selected periods of time are usually manageable because this correction factor can be applied to exclude the contribution of ENMs to the measured signal. There is also a possibility of combining various analytical methods so that reference methods (i.e., plate counting) are used along an alternative testing approach to correlate and validate the results.

#### Visualization and optical methods

Most methods for evaluating the viabilities and/or metabolic activities of microbes are based on the principle of optics: visualization, microscopy, imaging, fluorescence, spectrophotometry or their combinations. Such analytical methods are summarized in Table 1. Here, visualization and optical methods, including plate counting, disc diffusion, microscopy, fluorescence and optical density (OD), are reviewed, mainly based on their read-out features and applications. We also discuss some classical examples in more detail, including various special processes with essential pretreatments before measurements. We refer readers to original publications for unabridged statements.

**Table 1:** Analytical methods for assessing the antimicrobial activity of ENMs in complex matrices based on visualization and optical instruments

Method	Matrix	ENMs	Microbe(s)	Toolbox	Direct/indirect	Time needed	MIC detected	Principle, steps and features	Cost	Refs.
Plate counting	*Cotton fabrics*	Si	*E. coli*, *S. aureus*	Incubator, vortex meter, Petri dish	Direct number of viable microbes	~ 50 h	NM	Culture-dependent; assessing antimicrobial activity via the results of microbial viability; requiring treatment of sample before measurement	Low	[33]
	Chicken	Berberine- cinnamic acid				~ 25 h				[34]
	Sediment	Ag	Microbial community			> 48 h				[35]
	Estuarine water	TiO_2_	*E. coli*			~ 25 h				[29]
	Estuarine water	ZnO	Microbial community			~ 25 h				[36]
Disc diffusion	Cotton fabrics	CuO	*B. subtilis, E. coli, *etc	Incubator, caliper, Petri dish	Indirect	24 h	No	Culture-dependent; semi-quantitative evaluation via the zone of inhibition	Low	[37]
	Soil	Ag	Bacterial community							[38]
Microscopy	Seawater	Ag	Microbial community	Epifluorescence microscopy	Direct	NM	NM	Culture-independent; counting microbes using epifluorescence microscopy after stain with eye; assessing antimicrobial activity via the results of microbial viability	Medium	[39]
Fluorescence- imaging	Saliva	GO, Ag	Microbial community	Confocal laser scanning microscopy, live/dead bacterial viability kits	Direct	NM	No	Culture-independent; combination of viability staining with confocal laser scanning microscopy and detailed image analysis; assessing antimicrobial activity via the results of microbial viability	Medium	[40]
	Sludge	TiO_2_	Bacterial community		Direct					[41]
Fluorescence- high throughput screening	Lake water	TiO_2_	*E. coli*	Live/Dead BacLight kit, microplate reader	Indirect	~ 1 h	IC_50_ value	Culture-independent; staining live and dead cells with fluorescent dye; high throughput screening detection using microplate reader; assessing antimicrobial activity via the results of microbial viability	Medium	[42]
	Lake water	TiO_2_	Microbial community		Indirect		NM			[43]
	Lake water	CuO	*E. coli*		Indirect		NM			[44]
Fluorescence- ATP-based	Lake water	TiO_2_, Ag	*E. coli*	Microplate reader, BacTiter-Glo ATP assay kit	Indirect	~ 1–4 h	NM	Culture-independent; quantifying bacterial ATP by measuring luminescence signal intensity to show bacterial activity; assessing antimicrobial activity via the results of bacterial activity	Medium	[45]
	River water	ZnO, Fe_2_O_3_	*B. subtilis*		Indirect		NM			[46]
	Lake water	CuO	*E. coli*		Indirect		NM			[44]
Fluorescence-resazurin -based	Sludge	GO	Microbial community	Microtiter plate reader, metabolic assay kit	Indirect	~ 6 h	NM	Culture-dependent; staining cell with resazurin; assessing antimicrobial activity via the results of microbial activity	Medium	[47]
Fluorescence-flow cytometry	Sludge	TiO_2_	Bacterial community	Flow cytometer, ultrasonifier, filter	Indirect	NM	No	Culture-independent; distinguishing and quantifying live/dead cells with flow cytometer after staining; providing morphometric and functional properties of microbes; requiring detectable cells suspension; assessing antimicrobial activity via the results of microbial activity	High	[41]
	Seawater	Ag	Microbial community	Flow cytometer, filter, DNA dye		~ 1 h	NM			[48]
	Saliva	GO, Ag	Microbial community			NM	NM			[40]
	Lake water	Ag	Bacterial community			NM	NM			[49]
OD	Soil	TiO_2_, Ag, ZnO	Bacterial community	Microplate spectrophotometer, shaker, incubator	Indirect	A week^a^	NM	After pretreatment, nondestructively quantifying cells in transparent liquid solution with spectrophotometer based on the light scattering or/and absorption; assessing antimicrobial activity via the results of microbial activity	Medium	[50]
	Creek water	Ag, ZnO	Microbial community	UV–Vis spectrophotometer	Indirect	NM	NM			[51]
	*Cotton fabrics*	CuO	*B. subtilis, S. aureus, E. coli, P. aeruginosa*	Incubator, spectrophotometer	Indirect	~ 24 h	NM			[52]

*NM* not mentioned

^a^For individual measurement, the response time < 1 s

##### Plate counting

**Fig. 1:**
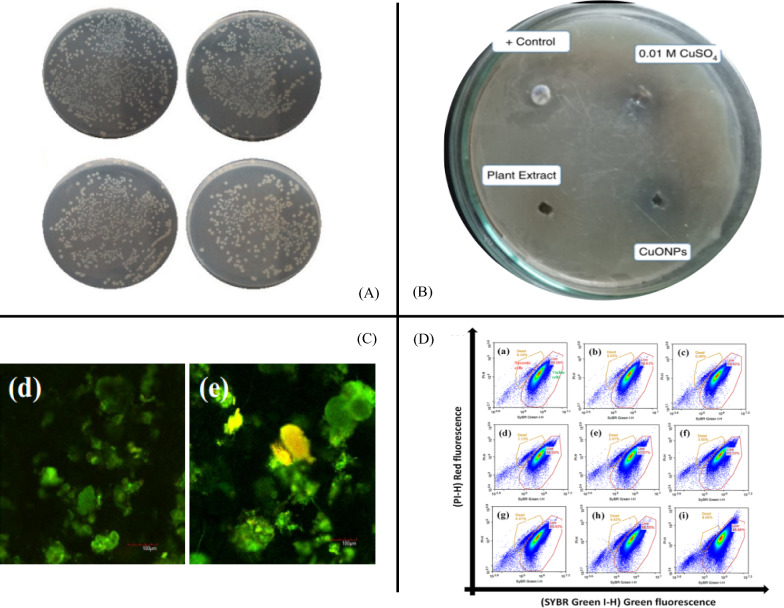
**A** Effect of berberine-cinnamic acid nanoparticle-modified packaging films on *E. coli* and *S. aureus* were characterized via a plate-counting method [34]. **B** Disc diffusion image of the antimicrobial assessment of CuO nanoparticles against bacterial species [37]. **C** Confocal laser scanning microscopy images of bacterial cells in activated sludge after an exposure to TiO_2_ nanoparticles: **d** 1 mg/L Ru-sun and **e** 1 mg/L An-sun [41]. **D** Flow cytometry cytogram of activated sludge cells stained with SYBR Green I + PI [41]

Plate counting, i.e., culturing and colony counting, is a well-established culture-dependent method for qualitative investigation of microbes. It is often used to measure the microbial viability after an exposure to target ENMs in complex matrices via live cells [29, 33–36]. A classical operation is given by Kusi et al. [35]: Treated sediment is added to Milli-Q water in centrifuge tubes and vortexed to detach microbes from the sediment particles. The tubes remain undisturbed for 30 min to allow sediment particles to settle and leave detached microbes in the supernatant. The supernatant is then diluted and inoculated into agar broth in Petri dishes. The plates are incubated at a desired temperature for 48 h, and colonies are counted at the endpoint. Figure 1A shows typical pictures of such a characterization [34].

The plate counting method is sensitive, easy and requires no expensive instruments. However, it requires relatively long culture times to allow the microbes to multiply sufficiently to form visible colonies. The manual readout is vulnerable to human error, less accurate and labor-intensive [31]. It may produce improper results during the evaluation of highly aggregated microbial cells [53]. Furthermore, despite the high recovery of bacteria (85–93%) from the original freshwater sediment via separation methods [54], the accuracy and reproducibility are still questionable. Thus, plate counting has largely been replaced when measuring the MICs of ENMs against microbes in complex matrices.

##### Disc diffusion

Disc diffusion is low cost, simple, flexible and easy to interpret [38, 53]; it is popular for assessing in vitro antimicrobial activity of ENMs in laboratory media [15, 55] as well as in complex matrices [37, 38]. For example, Turakhia et al*.* [37] used this method to assess the antibacterial activity of CuO nanoparticles modified on cotton fabrics. Briefly, cotton fabric samples modified with CuO nanoparticles were planted on an agar plate containing LB agar medium. The plates were inoculated with bacteria and incubated for 24 h. Zones of inhibition were then measured. The diameter revealed the sensitivity of microbes to the incorporated CuO nanoparticles (Fig. 1B). In general, to accurately assess the antimicrobial activity, aseptic instruments and materials must be used to prevent any false-negative results due to unwanted microbes [53].

The use of the disc diffusion method for assessing the antimicrobial activity of ENMs is questionable because the low diffusivity of materials practically prevents them from penetrating through the culture media. Kourmouli et al*.* [55] found that the disc diffusion method did not show any antibacterial effects of Au nanoparticles due to their negligible diffusivity through the culture media. In contrast, Ag nanoparticles exhibited a strong antimicrobial activity because the antimicrobial behavior was attributed to the ions that they release, thus dissolving upon oxidation and dilution in aqueous solutions. Cavassin et al*.* [15] reported that the diffusion method could be used as a screening test rather than as a reference test.

##### Microscopy

Microscopy is popular because it can visualize structural details. Further, microscopy can characterize the effect of antibiotics by counting cell numbers and morphologies [32, 56]. Echavarri-Bravo et al*.* [39] assessed the results of ENMs-microbe reactions in a microcosm experiment established with seawater and sediment samples. Ag nanoparticles and model marine bacteria were added to the water column. Total bacterial abundance in the water column was quantified at different time points by direct counts using epifluorescence microscopy and DAPI (4′, 6-diamidino-2-phenylindole) staining. This method requires staining of a relatively large number of microbial cells and is not precise in species identification [53]. Additionally, it has a low throughput in screening and requires harsh fixation, sometimes involving chemical cross-linking, drying, and high vacuum [16]. Low throughput and lack of standardization make inter-laboratory comparisons difficult, thus potentially leading to contradictory results [2].

##### Fluorescence-based methods

Microbial viability and/or metabolic activity can also be determined via fluorescence intensity. Fluorescence-based methods are more accurate than counting-based methods for determining adherent cells [57]. Table 1 shows various fluorescence-based methods used to assess the antimicrobial activities of ENMs: imaging (fluorescence confocal laser scanning microscopy), high throughput screening, flow cytometry, and ATP assays.

1. Imaging: Imaging characterizes the morphological changes in microbial cells. Confocal laser scanning microscopy rejects the light that does not come from the focal plane, thus enabling one to perform optical slicing and construction of three-dimensional (3D) images [58]. Confocal combines viability staining and detailed image analysis [40, 41].
  SYTO9 and propidium iodide (PI) are DNA dyes. Green fluorescent SYTO9 is membrane-permeable, whereas red fluorescent PI is not membrane permeable and quenches SYTO9 [40–44]. Li et al*.* [41] assessed the results of an ENMs–microbe reaction with imaging patterns by staining samples with SYTO9 and PI. After separating the microbes from the sludge, they performed live/dead staining according to the manufacturer’s instructions of the BacLight live/dead bacterial viability kits. Live bacteria were stained by SYTO 9 and fluoresced green; the dead bacteria were stained by PI and fluoresced red. The original floc structure was observed under a confocal laser scanning microscope, and representative images of bacterial cells in activated sludge after exposure to TiO_2_ nanoparticles are shown in Fig. 1C.
2. High throughput screening: A decrease in the ratio of fluorescent signals produced by SYTO9 (green) and PI (red) indicates a decrease in the number of live bacterial cells. The antimicrobial activity of ENMs can be accessed by reading the green-to-red fluorescence ratio of tested samples with a high throughput microplate reader [42–44]. In prior work, ENMs were added to lake water samples containing live bacteria. The mixtures were then incubated in the wells of clear bottom microplates. After the incubation, the SYTO9/PI mixture was added into each well of the microplates and mixed thoroughly. These microplates were then incubated for 15 min at room temperature in the dark followed by fluorescence measurements using a microplate reader. The green-to-red fluorescence ratio was calculated, and a calibration curve was obtained using bacterial mixtures with known percentages of live cells [42]. Chen et al*.* [44] reported no interference with BacTiter-Glo and BacLight assays in the case of testing 0.4 mg/L CuO nanoparticles and 2 mg/L TiO_2_ nanoparticles. However, the concentrations of microbes were not high enough for accurate assessments of natural lake or river water samples with this method. Consequently, pretreatment steps, e.g., centrifugation or filtration, were always needed before the incubation [43].
3. ATP-based method: ATP derives its inherent energy secondary to anhydride bonds connecting adjacent phosphate functional groups. It transports chemical energy within cells for various metabolic purposes. Live cells contain ATP, and ATP assays can measure live microbes quantitatively [59].
  Some research groups have used simple-to-use ATP-based assays to characterize the effects of ENMs on microbes in environmental water samples even at very low concentrations (e.g., < 20 μg/L [60]) where cell death was not apparent [44–46, 60]. Generally, BacTiter-Glo microbial cell viability assays can quantify ATP levels by measuring the luminescence signal intensity from the reaction of luciferin and ATP. Briefly, ENMs and liquid samples containing bacterial cells were incubated at room temperature. After the incubation, BacTiter-Glo reagent was mixed in each well, and the plate was covered with aluminum foil and incubated for 5 min before measuring luminescence with a microplate reader [44].
4. Resazurin-based method: The non-fluorescent phenoxazine dye resazurin can be taken up by live cells. The metabolic activity of microbial cells reduces resazurin to red fluorescent resofurin. The application of resazurin to check the microbial viability is of particular interest because it is non-toxic, easy to handle and requires relatively little preparation time [47, 61, 62]. Ahmed et al. [47] assessed the acute toxicity effects of graphene oxide on the wastewater bacterial community. Briefly, activated sludge samples were incubated with resazurin and exposed to different concentrations of graphene oxide in a 96-well flat bottom plate. The production of resofurin was quantified with a microtiter plate reader at 530/587 nm to assess the inhibition of metabolic activity by the ENMs.
5. Flow cytometry: Flow cytometry is useful in cell counting, cell sorting, chromosome preparation and biomarker detection in a stream of fluid [63]. It can help researchers detect almost all bacteria including non-culturable species, and reliably distinguishes and quantitates live and dead bacteria via a flow cytometer in a mixed population containing various bacterial types. Flow cytometry can provide morphometric and functional properties of the target microbes [31] by considering the light scattering and excitation/emission spectra of different fluorescent materials such as FRET dyes, fluorophores or fluorescent proteins [56]. This enables faster processing versus conventional methods—changes in physiological parameters are caused by ENMs and are faster than growth inhibition processes (1–2 h vs 16–24 h) [48]. This method has been applied to several microbial species and combinations of ENMs using various dye/fixation combinations [40, 41, 48, 49].
  It is difficult to identify individual microbial cells within non-fluid samples, e.g., sludge, by flow cytometry assays due to the variety, density, similarity of cells and non-biological particles. Thus, the preparation of detectable cell suspensions is an essential prerequisite. Li et al*.* [41] used dilution and sonication to completely disaggregate flocs and release free cells in the bulk liquid. The free cell suspension was filtered with a 20-µm membrane to eliminate coarse particles that might clog the nozzle of the flow cytometer. The resulting free cell suspension was then diluted with buffer to reach a suitable cell concentration for flow cytometry assays. Multicolor fluorescence combined with a dual-staining was used to distinguish subpopulations of bacteria after TiO_2_ nanoparticle exposure. The researchers identified live cells by staining with SYBR Green I/PI. The output resulted in a red versus green fluorescence cytogram showing single live (SGI^+^ PI^−^) and necrotic (SGI^−^ PI^+^) bacteria and lysed cell debris (SGI^−^ PI^−^) (minimal fluorescence) distributions (Fig. 1D). Of note, this work [41] did not describe the recovery of bacteria from the sludge nor the accuracy/reproducibility of the technique.
  Assessing the antimicrobial activities of ENMs in fluid samples with flow cytometry assays is easier than in non-fluid ones. After addition of Ag nanoparticles into natural seawater samples, Doiron et al. [48] counted bacteria in samples with an EPICS ALTRATM cell sorting flow cytometer with a 488-nm laser. Each sample was directly stained with SYBR Green I without any pretreatment steps for separation. Cells were incubated for 30 min at room temperature in the dark followed by flow cytometry analysis. Fluorescent beads were systematically added to each sample as an internal standard to normalize cell fluorescence emission. To quantify bacteria, the volume analyzed was calculated by weighing samples before and after each run. Total free bacteria were detected in a plot of green fluorescence recorded at 530 nm ± 30 nm versus side angle light scatter.
  Flow cytometry can quantify the antimicrobial activity of ENMs within a few hours, but it is rarely used due to inefficiencies with complex samples, especially meat, sludge, sediment, fabrics and soil. The staining inefficiency of dyes and autofluorescence are also key challenges [56].
6. OD method: In theory, when a light beam passes through a bacterial suspension in a clear solution, the scattered or absorbed light detected by a spectrophotometer correlates with bacteria density. Thus, the OD measurement is an alternative to nondestructively quantify target microbes [56, 64]. It is one of the most important and viable methods that can be potentially adapted into a high-throughput format for rapidly measuring bacterial cells after exposure to ENMs [50–52, 65]. This turbidity pattern can be easily adjusted for special culture conditions [66]. This phenotypic method strictly follows the Clinical and Laboratory Standards Institute (CLSI) and European Committee on Antimicrobial Susceptibility Testing (EUCAST) guidelines for AST, which is a major advantage [56]. A few multichannel automated machines, e.g., BD Phoenix, Vitek and BAXTER MicroScan, are commercially available for high throughput tests [32, 56, 67, 68]. For example, turbidity-based BD Phoenix can use up to 99 channels [69].

Recently, Chavan et al*.* [50] used the OD method to assess the effects of Ag nanoparticles, ZnO nanoparticles and TiO_2_ nanoparticles on bacterial communities in soil. Briefly, soil samples from a microcosm were shaken for 30 min in sterile saline. The solution was then further diluted and transferred to a plate for one week of incubation. The OD_590_ of the solution was measured with a microplate spectrophotometer every 24 h. Finally, the OD_590_ of the well was corrected by subtracting the control (no substrate) well at the same reading time. To investigate the bactericidal activity of CuO nanoparticles integrated with cotton fabrics, Shaheen et al*.* [52] developed a special OD method. Cotton fabrics coated with ENMs were immersed in liquid culture media, in which target bacterial cells were inoculated. The OD_630_ of the liquid culture media was then measured at a desired interval during the incubation. The bacterial growth in the nutrient medium was considered to be proportional to the OD values. Thus, the antimicrobial activity of CuO nanoparticles was calculated after an incubation of 24 h.

There are several important issues when using the OD method to assess the antimicrobial activity of ENMs in complex matrices: (1) The OD method is not suitable for low concentrations of bacteria [56]; (2) It cannot distinguish between live cells and dead cell debris [31]; (3) It is limited by cells forming chains, clumps (e.g. *Streptomyces koyangensis*), filaments, or aggregates; and is difficult to perform in complex media that can lead to light scattering or absorption and thus interference [39, 70]; (4) It is almost impossible to separate ENMs from biological samples without disturbing cell viability [65]; and (5) Scattering and absorbance from ENMs (e.g., arginine-functionalized gold composite nanoparticles [62]) act as interferences that complicate quantitative analysis [65, 71]. Pan et al*.* [31] commented that OD measurements were the most unreliable method to quantify the bacteria in the presence of ENMs.

We note that due to the unique physicochemical properties and increased reactivity of ENMs, there is a high potential for these materials to interfere with almost all kinds of spectrophotometric and spectrofluorometric measurements, thus leading to data artefacts and subsequent incongruent estimations of antimicrobial activities [72].

#### Molecular test-based methods

Molecular tests for antimicrobial activity research utilize molecular markers that are indicative of the presence of microbes and/or resistance. The vast majority of molecular tests in this area use quantitative and qualitative nucleic acid and protein markers via PCR, sequencing, metagenomics analysis and enzymatic viability analysis. Versus culture-based methods, the major advantage of molecular-based methods is that they reduce turnaround times for the culture step [67, 73]. Furthermore, they are particularly popular where non-culturable or slowly growing microbes are involved [67]. These assessments of antimicrobial activity are indirect because these values are calculated from molecular marker responses rather than from the quantitative and/or qualitative values of microbes.

##### Nucleic acid analysis

Developments in DNA/RNA analysis technologies, such as PCR, qPCR, RT-PCR, and high-throughput sequencing, have facilitated rapid microbe identification and characterization in genomes and metagenomes. They provide opportunities for rapid determinations of cultivable and uncultivable microbes from complex matrices. Table 2 describes nucleic acid analysis to assess the antimicrobial activity of ENMs in fluids, semi-fluids, and non-fluid matrices.

**Table 2:** Assessing the antimicrobial activity of ENMs in complex matrices based on nucleic acid analysis

Matrix	ENMs	Microbe(s)	Toolbox	Detection time	Principle, steps and features	Refs.
Estuarine water	TiO_2_	*E. coli*	RNA extraction kit, cDNA synthesis kit, centrifuge, PCR, electrophoresis, spectrophotometer, SPSS software	~ 5 h	Separating RNA; PCR amplification; calculating expression of special genes; assessing antimicrobial activity based on the change of special genes	[29]
Sludge	Ag	Ammonia-oxidizing bacteria, archaea	Centrifuge, shaker, electrophoresis, PCR, spectrophotometer, PCR purification kit, PCR detection system	> 5 h	Extracting DNA; qPCR amplification; calculating amoA gene abundances; assessing antimicrobial activity based on the change of amoA gene	[74]
Sludge	ZnO, Ag	Bacterial community	Centrifuge, dismenbrator, shaker, electrophoresis, PCR, spectrophotometer, cDNA synthesis kit, PCR purification kit, genetic analyzer, GeneMapper software	> 24 h	Separating RNA; amplifying bacterial 16S rRNA gene using cDNA; quantitative and qualitative analysis of bacterial relative abundance; assessing antimicrobial activity based on the change of biomass and relative abundance	[75]
Soil	Au et al. ^a^	Bacterial community	Soil DNA isolation kit, centrifuge, electrophoresis, PCR, DGGE system, molecular image FX apparatus, Gelcompar software package, PRIMER 5 software package	> 6 h	Extracting DNA; amplifying bacterial 16S rRNA gene; a second PCR with DGGE primers using amplicons obtained from the first PCR; quantitative and qualitative analysis; assessing antimicrobial activity based on the change of the bacterial diversity	[76]
Sediment	Pd	Bacterial community	Soil DNA isolation kit, centrifuge, electrophoresis, PCR, shaker, DGGE system, molecular image apparatus, Gelcompar software package,	~ 5 h	Cell lysis; DNA extraction and purification; amplifying bacterial 16S rRNA genes; calculating relative abundance of genes via DGGE analysis; assessing antimicrobial activity based on the changes of the bacterial structural and diversity	[77]
Seawater	Ag	Bacterial community	Filter, PCR, MinElute columns, 4001-Rev-B DGGE system, molecular image apparatus, Phoretix 1D Pro software	~ 5 h	Cell collection and lysis; DNA extraction; amplifying bacterial 16S rRNA genes; calculating bacterial richness via DGGE analysis; assessing antimicrobial activity based on the changes of the bacterial structural and diversity	[48]
Seawater	Ag	Bacterial community	Lyzozyme and proteinase K kit, centrifuge, electrophoresis, PCR, PCR purification kit, genetic analysis system and software package	> 5 h	Extracting DNA; amplifying bacterial 16S rRNA gene; digesting PCR products; calculating relative abundance of genes; assessing antimicrobial activity based on the changes of the bacterial relative abundance	[78]
River water	ZnO, Ag, TiO_2_	Bacterial community	Filter, DNA extraction kit, PCR, spectrophotometer, HotStarTaq plus master mix kit, molecular research^b^, BLAST database, Mothur software	> 5 h	Extracting DNA; purification; amplifying bacterial 16S rRNA gene; calculating relative abundance and diversity of genes via sequencing analysis; assessing antimicrobial activity based on the changes of the bacterial relative abundance and diversity	[79]
Lake water	TiO_2_	Bacterial community	DNA purification kit, PCR, electrophoresis, Mothur software, Roche 454 FLX sequencer, NCBI database	> 4 h		[43]
Wastewater	Ag	Bacterial community	DNA spin kit, PCR, electrophoresis, PCR purification kit, sequencing kit, sequencer, Ribosomal database	> 6 h		[80]
Creek water	Ag, ZnO	Bacterial community	DNA isolation kit, qPCR, MR sequencing machine, Ribosomal database	> 5 h		[51]
Soil	Ag	Bacterial community	DNA isolation kit, 16S metagenomics kit, fragment library kit, ion express barcodes, electrophoresis, Bioanalyzer, 1000 DNA kit, ion OneTouch 400 template kit, OneTouch ES instrument, Ion Torrent™ PGM, PGM™ sequencing 400 kit, Torrent-Suite software	NM	Extracting DNA; amplifying bacterial 16S rRNA gene; purification; enrichment; calculating diversity and community structure of genes via sequencing analysis; assessing antimicrobial activity based on the change of the bacterial diversity and community structure	[81]
Soil	Ag	Bacterial community	Soil DNA isolation kit, centrifuge, electrophoresis, qPCR, SYBR-Green qPCR master-mix	NM		[82]
Sludge	Au	Bacterial community	DNA isolation kit, HiSeq, PCR, Genomics Research Laboratory, metagenomics analysis server, M5RNA database, SEED Subsystems database, Comprehensive Antibiotic Resistance Database, Green Genes55 database	NM	DNA extraction; amplifying bacterial 16S rRNA genes; calculating genes abundance via DNA sequencing and metagenomics analysis; assessing antimicrobial activity based on the change of the bacterial relative abundance	[83]
Sludge	MnO_2_	Bacterial community	Soil DNA kit, centrifuge, electrophoresis, spectrophotometer, PCR, HTS MiSeq system	NM	DNA extraction; amplifying bacterial 16S rRNA genes; calculating bacterial relative abundance via DNA sequencing; assessing antimicrobial activity based on the change of bacterial communities	[84]
Sludge	ZnO, TiO_2_	Bacterial community	Only qPCR and HTS MiSeq system are mentioned	NM		[85]
Natural brackish water	Ag	Bacterial community	Filter, DNA spin kit, electrophoresis, NanoDrop, HTS MiSeq system, PCR, Ribosomal database, Mothur software	NM	Cell collection and lysis; DNA extraction; amplifying bacterial 16S rRNA genes; calculating genes relative abundance via DNA sequencing; assessing antimicrobial activity based on the change of bacterial communities	[86]
Sediment	TiO_2_, CeO_2_	Bacterial community	Tissue DNA kit, qPCR, NanoDrop, HTS MiSeq system, QIIME, RDP Classifier software	NM	DNA extraction; amplifying bacterial 16S rRNA genes; calculating bacterial relative abundance via DNA sequencing; assessing antimicrobial activity based on the change of bacterial communities	[87]
Sediment	CuO	Bacterial community	Plastic corer, Tissue DNA kit, PCR, HTS MiSeq system, Ribosomal database, Mothur software	NM		[88]
Sediment	Fe_3_O_4_, MWCNT	Bacterial community	Soil DNA kit, PCR, PCR purification kit, HTS MiSeq system, SILVA and QIIME software	NM	DNA extraction; amplifying bacterial 16S rRNA genes; calculating genes relative abundance and diversity via DNA sequencing; assessing antimicrobial activity based on the change of bacterial communities	[89]
Wetland	Ag	Bacterial community	Soil DNA kit, PCR, PCR purification kit, HTS MiSeq system, QIIME software	NM		[90]
Sludge	NiO	Bacterial community	Soil DNA kit, NanoDrop spectrophotometer, PCR, HTS MiSeq system, CANOCO software	NM	DNA extraction; amplifying bacterial 16S rRNA genes; calculating bacterial richness and diversity via DNA sequencing; assessing antimicrobial activity based on the change of bacterial communities	[91]
Sludge	Cu	Bacterial community				[92]
Sludge	ZnO, TiO_2_	Bacterial community	Soil DNA kit, electrophoresis, PCR, HTS MiSeq system, SILVA and QIIME software, NCBI database	NM	DNA extraction; amplifying bacterial 16S rRNA genes; calculating genes relative abundance and diversity via DNA sequencing; assessing antimicrobial activity based on the change of bacterial communities	[93]
Sludge	TiO_2_, Fe_3_O_4_	Bacterial community	Soil DNA kit, electrophoresis, PCR, HTS MiSeq system, QIIME software	NM		[94, 95]

*NM* not mentioned, *HTS MiSeq system* High throughput sequencing based on the Illumina MiSeq system

^a^Au, TiO_2_, carboxylmethyl-cellulose, polyethylglycol, didodecyl dimethylammonium bromide, monoolein/sodium oleate, titanium silicon oxide, CdSe/ZnS quantum dots and Fe/Co magnetic fluid

^b^One of the next-generation DNA sequencing systems

A small minority of the gene analysis-based methods are used to assess the antimicrobial activities of ENMs by quantifying single microbial species [29] or special genes [74]. Herein, we select Qin et al*.* [29] as a representative example. Briefly, *E. coli* in aquatic environment samples were homogenized, and the total RNA was extracted using an RNA extraction kit and quantified using a UV–VIS spectrophotometer. The RNA quality was monitored with agarose gel electrophoresis. The cDNA was synthesized using a cDNA synthesis kit. A qPCR assay was performed with 16S rDNA primers as a housekeeping gene and served as an internal control for tested gene expression analysis. Gene expression was calculated using the 2^−ΔΔCt^ method. Significant differences in the tested gene expression were assessed by one-way analysis of variance (ANOVA) followed by Tukey's HSD test using SPSS software.

Most gene analysis-based methods analyze whole genes extracted from complex matrices [43, 48, 51, 75–95]. Researchers obtain detailed information about bacterial community structure, relative abundance and/or diversity via an amplification and analysis of 16S rRNA genes. They then infer the antimicrobial activities of target ENMs against the entire bacterial community. In general, in the case of fluid complex matrices, the experimental operation consists of separation of microbial cells (by centrifuge or filter), a cells lysis step, extraction and purification of genomic DNA, amplification and final data analysis via a computer and special software(s). Here, we selected Doiron et al*.* [48] to show the procedure with a final DGGE DNA analysis. Briefly, seawater samples were filtered with a polycarbonate membrane. Total DNA was extracted from the filter after a cell lysis step. PCR amplification of the 16S rDNA gene was performed using universal primers. Amplicons were then purified with special columns and stored until DGGE analysis. DGGE was performed using a DGGE-4001-Rev-B system. Gels were then stained with a half-diluted solution of SYBR Green I for 1 h and photographed under UV light. Finally, DGGE profiles were analyzed using Phoretix 1D Pro software to show bacterial richness. From the information on bacterial richness, the authors inferred the effect of Ag nanoparticles on the bacterial community structure. We also highlight Londono et al*.* [79] to illustrate sequencing. Here, microbes were filtered from river water and subjected to DNA extraction. The DNA concentration and quality were checked with a Nanodrop spectrophotometer. PCR amplification of the 16S rDNA gene was performed using the HotStarTaq Plus Master Mix Kit. Next-generation DNA sequencing was conducted by Molecular Research systems. An in-house proprietary analysis pipeline was used to process the sequence data. The remaining sequences were then denoised, and chimeras were removed using UCHIME implemented in Mothur software. The operational taxonomic units were taxonomically classified using BLASTn against a database derived from NCBI and RDPII and compiled into each taxonomic level into both “count” and “percentage” files. Sequence counts by taxa were further analyzed using R—a free software environment for statistical computing and graphics. Microbial community differences between groups were tested for significance using the two-factor Adonis function of the vegan package. Heat maps were created to illustrate microbial distribution in tested samples using hierarchical clustering of relative abundance. Species richness was determined by a genera or species taxa count in each sample. The Shannon index for species diversity in a given community for each tested condition was calculated using the diversity function in the Vegan package, and species evenness was calculated as the Shannon index divided by the natural logarithm of species richness.

A completed separation of live microbial cells is very difficult in non-fluid complex matrices (e.g., sludge and soil). Usually, chemical and biological reagents are directly added into the matrices to lyse microbial cells [84, 89–95]. The total DNA/RNA is then extracted from the complex matrices. A few commercial kits can facilitate this task. The remaining experimental operations (DNA purification, amplification, gene analysis) are similar to that for fluid matrices. We select Nuzzo et al*.* [77] as an example of the determination procedure with a final DGGE DNA analysis. Briefly, slurry samples were centrifuged and the water phase was discarded; metagenomic DNA was extracted from the wet sediment with a Power Soil DNA extraction kit. 16S rRNA genes of the bacterial community were amplified using PCR with special primers. DGGE of bacterial amplicons were performed with a D-Code apparatus with a denaturing gradient from 40 to 60% denaturant. Gels were stained with SYBR Green I and their image was captured in UV transilluminator with a digital camera. Community richness and organization indexes were calculated from DGGE image analysis.

Miao et al*.* [87] reported a typical procedure with a sequencing analysis for assessing the effect of ENMs on bacterial community in soil. Briefly, wet sediments were collected and frozen with ethanol and dry ice. Genomic DNA was extracted using a tissue DNA kit. The concentration of extracted DNA was measured with a NanoDrop and Pico Green assays. Subsequently, real-time qPCR was used to determine the copy numbers of the 16S rRNA gene of all bacteria in the sediment with the fluorescent dye SYBR green approach. The bacterial community was investigated by Illumina high-throughput sequencing. The raw data were saved as paired-end fastq and raw fastq files were demultiplexed using QIIME. After removing the barcodes and primers, the data were subsampled to 13,876 sequences per sample to avoid biases related to unequal numbers of sequences. The normalized samples were then individually classified and analyzed by the RDP Classifier.

RNA can also be used as a biomarker. For instance, Chen et al*.* [75] extracted total RNA from the centrifuged sludge pellets. They performed a reverse transcription PCR with specific primers to obtain the cDNA before amplification of bacterial 16S rRNA genes.

Here, we emphasize a few important issues in gene analysis research.

1. Not all genetic materials extracted from natural complex samples are sourced from live cells. There may be some environmental DNA (eDNA), i.e., the genetic material present in environmental samples, such as sediment, water, and air, including whole cells as well as extracellular DNA released from dead cells [96]. eDNA is reported to persist for days, weeks or years in environmental samples [97]. PCR assays cannot discriminate between live and dead cells [98]. Future work should accurately quantify gene copy number from *live* microbes perhaps via coexisting extracellular DNA in the complex matrices [99]. Propidium monoazide can intercalate into double stranded DNA and form covalent linkages, thus resulting in chemically modified DNA that cannot be amplified by PCR [100, 101]. However, the intercalation requires exposure of blue light. Thus, the addition of propidium monoazide cannot be used in most complex matrices (e.g., sludge and sediment) because light cannot penetrate into the sample.
2. DNA and RNA can rapidly adsorb onto all known ENMs when entering complex physiological or ecological environments [102]. Thus, it is questionable to quantify the antimicrobial activities of ENMs via PCR analysis due to the interference of ENMs with PCR amplification [100]. Thus, inhibitory concentration values based on the concentration–response relationship cannot be derived via gene analysis [81].
3. The cost is rather high due to the need for expensive reagents and machinery with specific maintenance conditions. Few units are available for point-of-care use. Thus, a transfer of samples is required.
4. Note that most of these gene analysis-based methods involve complex workflows (e.g., cell separation, cell lysis, DNA/RNA separation, genetic materials purification, amplification and sequencing analysis). Technological hurdles remain despite automation. Upstream sample processing is difficult to be automated [67]. These manual and semi-manual steps have a prerequisite of skilled personnel. Furthermore, these cumbersome steps cause slow turnaround times. For instance, DNA extraction and library preparation can still take up to 5 h prior to sequencing analysis [103].
5. One of the key prerequisites are prior sequence data of the specific target gene to estimate the changes of microbial diversity, community structure, richness and relative abundance based on the data of gene analysis [53]. This means that the reliability of the measurement depends on the perfection of professional databases rather than the method itself.

##### Protein analysis

Apart from nucleic acid markers, protein (enzyme)-based molecular signatures can also be used to assess the antimicrobial activities of ENMs in complex matrices via suitable readout approaches. This field has benefited from protein analysis, e.g., lactate dehydrogenase (LDH) kits are widely available to measure LDH released by damaged cells [72]. Table 3 summarizes protein analysis-based methods to assess the antimicrobial activities of ENMs in complex matrices, e.g., natural water, soil and sediment.

**Table 3:** Assessing the antimicrobial activity of ENMs in complex matrices based on protein analysis

Matrix	ENMs	Microbe(s)	Toolbox	Detection time	MIC detected	Principle, steps and features	Refs.
Sediment	Fe_3_O_4_, MWCNT	Bacterial community	Filter, UV–vis spectrophotometer, incubator	> 1 day	No	Mixing sediments with chemical reagents; calculating urease, DHA and PPO activity based on absorbance values of supernatant	[89]
Sediment	Ag	Microbial community	Multiskan ascent plate reader, vortexer, pipette, incubator	> 1 day	No	Mixing sediments with chemical reagents; calculating APA and β-GA activity based on absorbance values of supernatant	[35]
Soil	Ag	Nitrogen cycle microbes	Victor multilabel plate reader, incubator	NM	No	Mixing slurry with chemical reagents; calculating LAP activity based on fluorescence values of supernatant	[104]
Soil	Ag, Al_2_O_3_, SiO_2_	Microbial community	Filter, UV–vis spectrophotometer, incubator, shaker	> 1 day	No	Mixing sediments with chemical reagents; calculating urease and DHA activity based on absorbance values of supernatant	[105]
Soil	Ag			> 20 h	IC50	Mixing soil with chemical reagents; calculating DHA activity based on absorbance value of supernatant	[81]
Sediment	CuO		ELISA kit, microplate reader, incubator	> 3 h	No	Determining APA, β-GA and LAP activity with ELISA kit via absorbance analysis	[88]
Sludge	ZnO, TiO_2_		Centrifuge, LDH assay kit	NM	No	Separating enzyme from sludge by centrifuge; determining LDH activity with ELISA kit	[85, 93]
Sludge	TiO_2_		Sectrophotometer, water bath, shaker, LDH kit	NM	No	Mixing sludge with chemical reagents; determining ammonia monooxygenase, nitrite oxidoreductase, nitrite reductase, nitrate reductase, polyphosphate kinase and exopolyphosphatase activity based on absorbance values of supernatant; determining LDH activity with ELISA kit	[94, 95]
Sludge	ZnO		Sectrophotometer, incubator, shaker	NM	No		[106]
Wetland	Ag						[90]
						Assessing antimicrobial activity of target ENMs based on the changes of enzyme activity	

*DHA* dehydrogenase, *PPO* Polyphenol oxidase, *APA* alkaline phosphatase, *β-GA* β-glucosidase, *NM* not mentioned, *LAP* leucine aminopeptidase, *LDH* Lacate dehydrogenase

Enzymes have important biochemical and microbiological roles in natural matrices. Enzyme activity assays can measure the antimicrobial activities of ENMs. A common procedure was reported by Xu et al*.* [89]. Briefly, sediment samples were mixed with the needed chemical reagents for urease activity detection. Ultrapure water was added after incubation. The supernatant was filtered, and the ammonium concentration of the filtered extracts was determined by measuring the absorbance with a UV–vis spectrophotometer. The value of the enzyme activity was calculated with a working curve. Finally, the antimicrobial activity of target ENMs in complex matrices was evaluated based on the change of enzyme activities. Recently, reagent kits for facilitating these tasks have become commercially available [88, 94].

Protein analysis is less complicated than gene analysis, as it requires fewer reagents and instruments, thus leading to a medium cost. However, the stability of the results still depends on the operator skill level. In general, protein analysis lacks sensitivity because of the absence of an amplification step [53].

##### Common issues of molecular test-based methods

One of the primary issues impacting molecular tests may be the interference resulting from ENMs themselves [100], thus leading to data artefacts and subsequent incongruent estimations of antimicrobial activity. These inconsistent and/or inaccurate data make it difficult for regulators to establish guidelines and procedures, ultimately hindering our ability to predict how ENMs will affect organisms in complex matrices [72].

- These methods are relatively expensive and require cumbersome steps such as cell lysis, genetic materials/protein separation, purification and transfer. Even skilled professionals cannot completely avoid all objective and subjective errors.
- There are few studies on the recovery of the extraction of genetic materials and separation of protein from complex matrices. In theory, it is impossible to guarantee recovery and reproducibility. Therefore, these methods are rare in quantifying the inhibitory effect of ENMs against target microbes and microbial communities.
- It is impossible to perform on-line monitoring. Thus, molecular tests act as a powerful supplementary tool rather than replacing phenotypic susceptibility testing.

#### Mass test-based methods

The analysis of the microbial mass change resulting from exposure is an alternative method for assessing antimicrobial activities of ENMs. The microbial mass change can be inferred from the mass change of total DNA [104, 107], protein [49, 108] and other biomarkers (e.g., ergosterol [109]), which are separated from the tested complex matrices.

Grün et al*.* [104] assessed the effect of Ag nanoparticles on microbes in soil. They extracted and purified genomic DNA with a commercial soil kit to measure the change of microbial biomass. For the measurement of microbial biomass, 10 μL of DNA was transferred into the well of a 96-well microplate and shaken for 5 s before absorbance was measured at 260 nm. Das et al*.* [108] measured bacterial production from protein synthesis using ^3^H-leucine incorporation. Briefly, subsamples from the Ag nanoparticle addition experiment were incubated for 1 h with ^3^H-leucine. Incubations were stopped by the addition of formalin. Bacterial cells were harvested by filtration onto 0.22-mm polycarbonate membrane filters, and proteins were precipitated with repeated washing with tricholoracetic acid. The radioactivity of each filter was assessed by liquid scintillation counting and counts converted to micrograms C/L/d. Note that specific bacterial production estimates could not be determined. To the best of our knowledge, there are still no reports that directly measured microbial mass separated from tested matrices before and after exposure.

Versus molecular test-based methods, these methods are simpler and more cost-effective. However, the results also depend on operators’ skill level. In addition, we cannot find any critical analytical properties (e.g., recovery, sensitivity, accuracy and reproducibility) of these methods. In theory, it is hard to use them for quantitative analysis because it is impossible to guarantee recovery and reproducibility.

#### Respiration-based methods

Methods based on microbial respiration, including the heterotrophic respiration [39, 81, 87, 88, 110] and the metabolic quotient [111], have been reported for assessing antimicrobial activities of ENMs in soil and sediment. For instance, heterotrophic respiration of biofilm communities was measured in sediment cores using a DO sensor. The sensor was placed ~ 1 cm from the water/sediment interface, and oxygen concentrations were recorded after sealing the sediment core. The amount of consumed oxygen was calculated over time and averaged over all repeated measurements. The resulting values obtained were used to infer the effect of CuO nanoparticles against biofilm communities [88]. A basal respiration assay was performed in another report [111]. Pre-incubated test soil was added to sterilized glass vials prepared for four incubation periods; these were then capped with a rubber septum. At the time of sampling, the vials were sampled by removing 1 mL of headspace with an airtight syringe. The headspace sample was injected into a CO_2_/H_2_O analyzer and the time and resulting observed peaks were recorded to calculate the rate of CO_2_ released per gram of soil. The metabolic quotient (qCO_2_) was calculated as a ratio of respiration to biomass (qCO_2_ = respiration/biomass) to assess the effect of Ag nanoparticles on microbial activity.

**Fig. 2:**
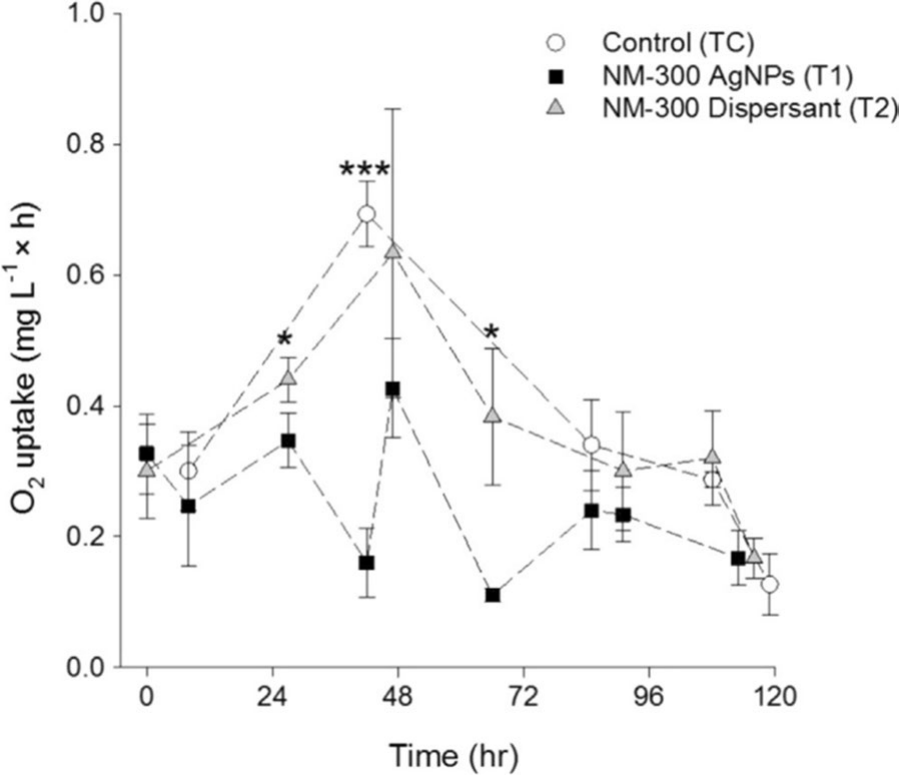
The O_2_ uptake changes of microbial communities during the course of the microcosm exposing to ENMs; results are expressed as the mean value of the O_2_ uptake ± S.D [39]

Respirometry assays can be applied to measure the O_2_ uptake of the entire microbial community inhabiting the matrices and can provide insight into the overall physiological status of the community including non-culturable aerobic bacterial groups. These assays can also provide information about the immediate bacterial response within 0.5 h of temporal resolution. Respirometry is simpler and more cost-effective than molecular and mass-based methods; however, it has low precision and reproducibility (Fig. 2) [39].

### On-line measurement

A common feature of all methods reviewed above is that they rely on off-line measurement. These methods cannot monitor microbial growth kinetics with high temporal resolution in the presence of ENMs due to the long measurement turnaround time. In realistic scenarios, microbes live in communities such as biofilms, mats, and flocs with an intricate structure created by a diverse consortium of bacterial, Archaean, fungal, and algal species attached to a substratum and embedded within a matrix of extracellular polymeric substances [112]. Measuring microbial viability and/or metabolic activity often requires sample pretreatment. These pretreatment steps (e.g., separating for gene analysis) might miss markers with a subsequent influence on the experimental results [113]. Therefore, common off-line endpoint measurements are insufficient for reliable assessments of the antimicrobial activities of ENMs in most complex matrices. On-line and real-time analysis of microbial susceptibility to ENMs will provide more information for an optimized method than static acquisition of single data points [114].

Monitoring the morphology changes of ENM-microbe interactions or microbial growth on-line is very difficult in complex matrices due to the difficulty of signal read-out. For instance, the accuracy of automated optical-based methods inevitably suffers from interferences from co-existing substances and bacterial clumps [62]. To date, there are no commercially available instruments and automated phenotypic methods to monitor microbial growth in complex matrices, e.g., soil, sludge, blood and food [2]. However, pioneering studies like luminescence- [115, 116] and electronic sensor-based methods [71, 117] have recently been reported (Table 4).

**Table 4:** Assessing the antimicrobial activity of ENMs via microbial growth curves generated by on-line monitoring the interaction processes

Method	Matrix	ENMs	Microbe(s)	Toolbox	Principle	Direct/indirect	MIC detected	Accuracy and precision	TROGC	Cost	Refs.
Luminescence	Wastewater	TiO_2_, Ag, ZnO	*P. putida*	SpectraMax M5 reader, microtitre plate, shaker, incubator	Using luminescent (switch-off) *P. putida* BS566::luxCDABE as bacterial bioreporter; generating bacterial growth curve by real time monitoring the emitted luminescence evolution with a spectra reader; evaluating antimicrobial activity of ENMs based on their effect on the growth curve	Direct	IC_50_	NM	15 min	Medium	[115]
		Ag						SD: ~ 30%	NM		[116]
Electronic sensor	Magnetic beads suspension	Se	*E. coli*, *S. aureus*	8 channel C^4^D	Automated phenotypic method; on-line monitoring capacitively coupled contactless conductivity changes of total media with electronic sensor to generate bacterial growth curve; assessing antimicrobial activity of ENMs based on their effect on the growth curve at each stage	Indirect	Yes	NM	1 min	< 1 $	[117]
	Modified river and sea water	Ag, Au		8 channel C^4^D				6, 5, 6 and 5 out of each 9 repeated measurements are the same MIC results; MICs within 3.50 ± 1.00 μg/mL	0.5 min	< 1 $	[71]

*TROGC* Temporal resolution of growth curves, *P*. *putida* BS566::luxCDABE was constructed by transposon (Tn5) mutagenesis based insertion of the full lux operon from *Photorhabdus luminescens* along with a kanamycin-resistance gene as a marker into the genome of a *P. putida* BS566 strain originally isolated from a wastewater treatment plant, *NM*  not mentioned

#### Luminescence-based methods

Mallevre et al*.* [115, 116] used the switch-off *Pseudomonas putida* (*P. putida*) BS566::luxCDABE bioreporter as a model bacteria to assess the antimicrobial activity of ENMs in wastewater. In brief, bacteria were pre-cultured overnight under shaking conditions in wastewater and then freshly diluted to reach a final concentration. Stock suspensions of Ag nanoparticles were serially diluted to give final tested concentrations. All wastewater samples were supplemented with D-glucose prior to use to ensure a consistent minimal amount of carbon source. Assays were conducted in black walled 96-well microtiter plates. Monitoring of the emitted luminescence evolution of *P. putida* was performed using a SpectraMax M5 reader in a kinetic mode for 2 h. Results were expressed in relative luminescence (% RLU) and plotted against time for selected conditions. Ag nanoparticle toxicity was expressed as IC_50_ at 1 h.

**Fig. 3:**
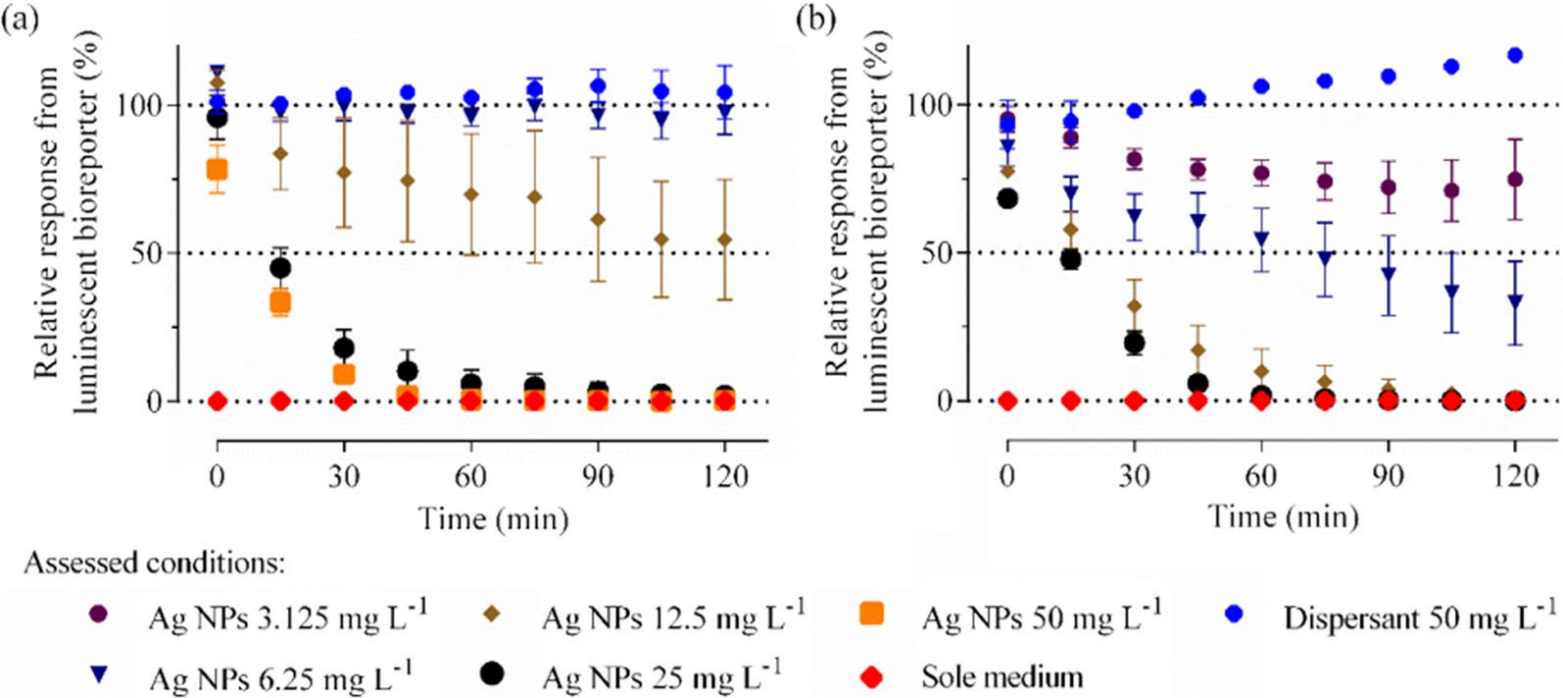
Real time monitoring of Ag nanoparticle toxicity in wastewater [116]. Relative luminescence output evolutions over time by *P. putida* BS566::luxCDABE when challenged up to 200 mg/L ENMs in **a** crude and **b** final wastewaters. Data are mean ± standard error of the mean (n = 4)

The operation of the luminescence-based method is easy because of the absence of sampling. No expensive instruments are needed other than the spectra reader. The growth curves provide detailed information on the bacteria inhibition of the ENMs at each growth stage, thus enabling users to directly read out IC_50_ values. However, the accuracy and precision are poor (see the error bar in Fig. 3) [116]. This method is thus limited to transparent liquid matrices.

#### Electronic sensor-based methods

**Fig. 4:**
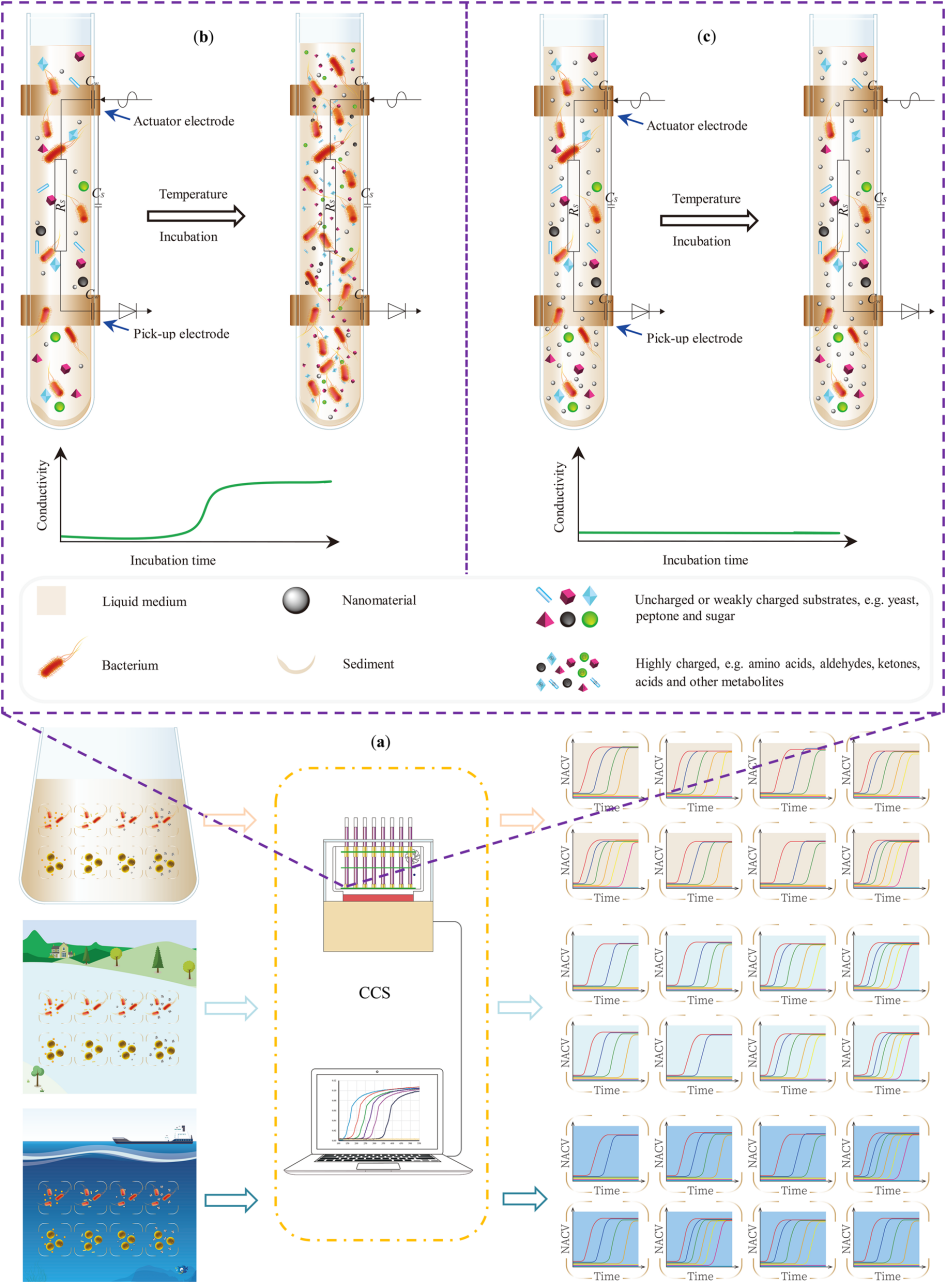
Schematic diagrams of the automated CCS method for determining the antimicrobial activity of ENMs [71]. In both simple and modified river and sea water, the growth kinetics of model bacteria (*E. coli* and *S. aureus*) were determined to generate growth curves that enable users to directly obtain MICs (**a**). Insert: Test tubes loaded with aqueous samples are inserted in the CCS. The capacitively-coupled contactless conductivities of the liquid samples are monitored on-line in a non-invasive way. When the concentration of target ENMs is below the MIC, a sigmoidal growth curve is generated by plotting conductivity values as a function of incubation times (**b**). No conductivity changes in the medium leads to a straight line when the concentration of target ENMs is equal or higher than the MIC (**c**)

Our group constructed an automated phenotypic method to directly determine the antimicrobial activity of ENMs by developing multi-channel contactless conductometric sensors (CCS) [71, 117]. The working window of the contactless conductometric sensor covers the conductivity range of simple laboratory solutions and common realistic aqueous samples. It allows simultaneous cultivation and on-line monitoring of the kinetic process of bacterial growth in the presence of ENMs and provides high temporal resolution growth curves. As such, the automated phenotypic method enables users to directly obtain accurate MIC (the principle is shown in Fig. 4). Briefly, to determine the bacteriostatic activity of ENMs, modified river or sea water samples containing *E. coli* or *S. aureus* cells were loaded in disposable testing tubes. The ENMs were added into individual tubes to make a series of concentrations. All tubes were then simultaneously inserted into the CCS. The capacitively coupled contactless conductivity of the aqueous media in each tube was collected at a rate of 0.5 min. A sigmoidal growth curve was then generated by plotting the conductivity values as a function of incubation time. The bacteria growth may be completely inhibited at high concentrations (≥ MIC) of ENMs, thus leading to a straight line [114]. This sensing method highlights the advantages of universality, simplicity and affordability and is a new field of analytical chemistry for determining the antimicrobial activity of ENMs. However, it should be validated with real samples before use to determine accurate MICs for risk assessments of ENMs in realistic scenarios.

## Emerging alternatives

In theory, the assessment of ENMs’ antimicrobial activity is similar to the AST. However, not all AST methods are suitable for this task. For instance, Kourmouli et al. [55] found that disc diffusion susceptibility testing was not suitable for assessing the antimicrobial activity of Au nanoparticles. Likewise, many analytical methods have been used to characterize the results of ENMs-microbe interactions [2]. Of these, only a few are suitable for determining the results of interactions in complex matrices, especially non-fluid samples mainly due to nonhomogeneous conditions [113]. The need for sample pretreatment contributes substantially to the variation of readout, thus increasing measurement errors.

In this context, we provide some promising recommendations for measuring microbial viability and/or metabolic activity in the presence of ENMs in complex matrices. These emerging techniques have the likelihood to yield fast, reliable and reproducible data on ENMs-cell responses in vitro. The criterion for our recommendations is the theoretical feasibility and practical value rather than the number of papers published per technique.

### On-line monitoring-based methods

#### Hyperspectral imaging

**Fig. 5:**
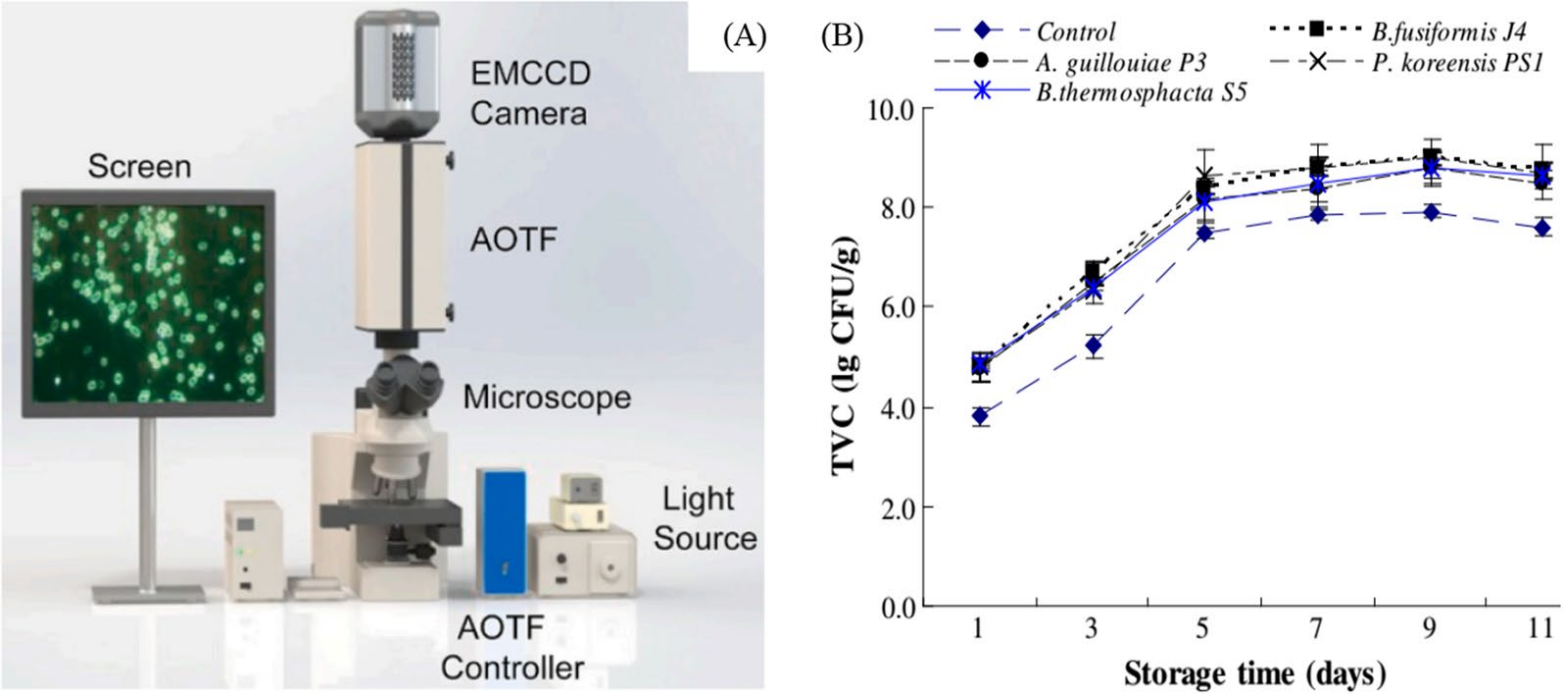
Hyperspectral imaging system [118] **A** and the change of total viable count of bacteria in pork meat with storage time monitoring with the hyperspectral imaging system (**B**) [120]

Hyperspectral imaging (Fig. 5A [118]) analyzes a wide spectrum of light instead of only assigning primary colors (red, green, blue) to each pixel. The light striking each pixel is broken down into many different spectral bands to provide more information on the sample. In dark-field microscopy, targets are uniquely identified by light scattering patterns of cells, thus providing high signal-to-noise ratios to acquire cellular images from the background [119]. On a microscopic level, cellular images are generated with a hyperspectral microscope and serve as the bacteria’s “fingerprint”; theoretically, any pathogen can be detected with a spectral fingerprint using hyperspectral images once a reference library from pure bacterial isolates has been created. This technique can directly detect live bacteria in complex matrices such as chicken rinsate [119] and pork meat [120]. Data on total viable counts of bacteria in pork as a function of storage time were generated using a hyperspectral imaging method (Fig. 5B). Hyperspectral imaging has also been used to analyze the kinetics of Ag ion leaching from nanoparticles [121].

#### Raman spectroscopy

Raman spectroscopy, especially surface-enhanced Raman spectroscopy (SERS), is an attractive approach for biological sensing due to its high sensitivity, real-time response, and capacity for molecular fingerprinting [122–126]. It provides a simple and even quantitative manner to monitor the microbial activity and the associated responses of activity to antibiotics [126].

Weidemaier et al*.* [124] reported an approach for the real-time detection and identification of pathogens in complex culture matrix. In brief, SERS-labeled immunoassay nanoparticles were present in the cultural enrichment vessel, and the signal was monitored in real-time through the wall of the vessel during culture. This continuous monitoring of a specific microbe loaded throughout the enrichment process enabled rapid and hands-free detection without interfering with microbial growth, thus significantly reducing the risk of contaminating the surrounding environment. Wang et al*.* [125] reported using SERS for detection of both volatile and nonvolatile metabolites. This approach was used to quantify bacterial growth. The time-dependent SERS signal of the volatile metabolite dimethyl disulfide in the headspace above bacteria growing on an agar plate was detected and quantified on-line.

Raman spectroscopy is feasible for assessing the antimicrobial activity of ENMs even in complex matrices; it does not interfere with microbial growth. However, the robustness of the SERS signal is dependent on the ability to concentrate the plasmonic particles in the area of the laser. For certain matrices with large particulates, care must be taken in the design of the instrument and the magnetic pelleting system to ensure that these particulates do not interfere with reproducible concentration of the plasmonic particles.

#### Contactless resonator sensor

Similar to contactless conductometric sensors [71, 117, 127], contactless resonator sensors are popular because they non-invasively monitor bacterial growth [128–130]. Generally, the resonance frequency of an immersed magnetoelastic sensor is measured through magnetic field telemetry; thus, these changes are mainly in response to microbial adhesion. In turn, the decrease allows one to calculate the microbial concentration. The lack of any physical connection between the measurement sensor and the culture medium facilitates aseptic operation and avoids electrode deterioration, thus making the platform ideally suited for on-line monitoring. These methods have been successfully used for rapid and real-time monitoring of bacterial growth against antibiotics in solid growth medium [129].

#### Electrochemical sensing

Electrochemical methods offer relatively simple instrumentation, easy miniaturization, cost-effectiveness and easy automation of measurements. They are thus interesting tools for monitoring microbial growth [64, 131]. Impedance/capacitance [132–137] and potentiometry (especial pH-metry) [138–140] are usually applied because the growth of microbes changes the electrical properties and the strength of the acids. Indirect conductivity can be used to measure CO_2_ production as an indicator of microbial growth [141, 142].

**Fig. 6:**
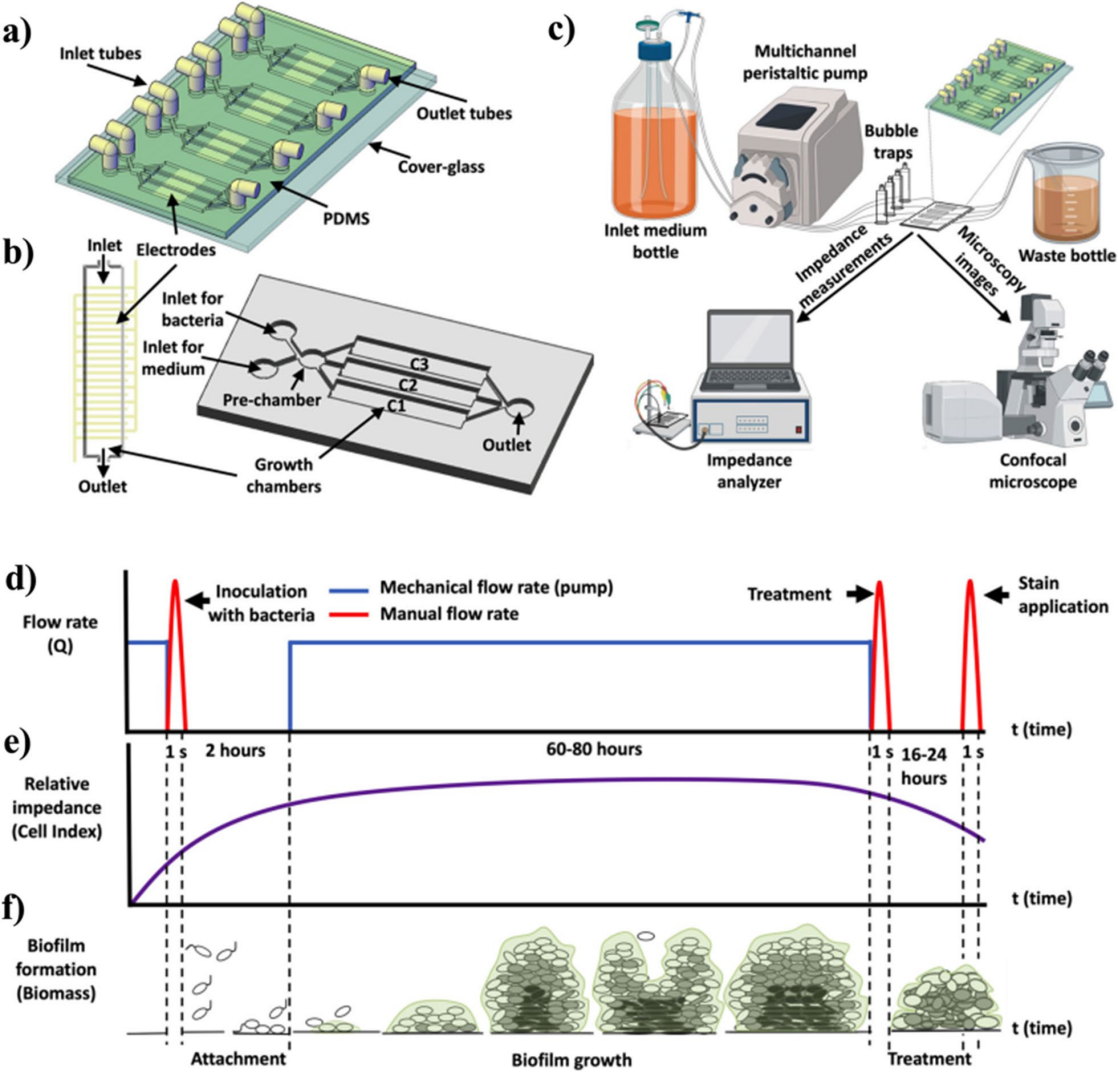
Bio-film-chip 3D view (**a**), 3D view of one chamber with the electrodes and one set of 3 chambers (**b**), experimental setup (**c**), changes in the mechanical flow rate and the manual flow rate (**d**), expected relative impedance (**e**), and biofilm formation over time (**f**) [136]

Impedance/capacitance has been used to monitor bacterial growth on-line in both ideal liquid culture media [132, 133] and complex matrices [134–137]. These methods can also perform ASTs in nonhomogeneous media [135, 136]. For instance, Blanco-Cabra et al*.* [136] presented a microfluidic platform with an integrated impedance sensor. This device allowed an irreversible and homogeneous attachment of bacterial cells of clinical origin even directly from clinical specimens. The resulting biofilms were monitored by electrical impedance spectroscopy, thus providing a suitable protocol to study polymicrobial communities as well as to measure the effect of antimicrobials on biofilms without introducing disturbances, thus better mimicking real-life clinical situations (Fig. 6).pH-metry is independent of the type of microbes as well as the nature and chemical properties of the substrate used to grow the cells. It has been used to monitor microbial fermentation [138] and can analyze microbial viability in complex solutions as demonstrated in spiked milk and human whole blood [139].

Actively growing bacteria releases CO_2_ metabolites that can change the conductivity of interlinked KOH agar solution. Thus, conductimetric technology can be used to indirectly monitor microbial growth. The Callanan group introduced an indirect conductivity method to study the bacterial growth in complex food matrices [141, 142]. The linearity of conductivity responses in selected food products was investigated with good correlation (*R*^2^ ≥ 0.84) between inoculum levels and times to detection.

Despite these significant advantages, contact electrochemical methods suffer from electrode deterioration and nonspecific binding (the working electrodes must be in galvanic contact with the medium) [64]. These issues should be considered when researchers use contact electrochemical methods for determining the antimicrobial activity of ENMs. Such invasive measurements may result in erratic results that decrease the accuracy [64, 143]. Prior work [144] offers key tips to solve electrode fouling.

#### Mass spectrometry

Different species of microbes usually live together in real samples or even in culture medium. Mass spectrometry is a promising method for monitoring the characteristics of different species growth because the microbe’s metabolism leads to the production of highly diverse multiple volatile organic compounds [99, 145]. Sovová et al*.* [99] monitored population dynamics in concurrent bacterial growth using mass spectrometry quantification of volatile metabolites. The concentrations of volatile metabolites were measured in the headspace of the individual species, and their mixtures were continuously monitored for 24-h periods. The results demonstrated that this method could be utilized to monitor bacterial proliferation in real time without interfering with the living organisms. Mass spectrometry was successfully used for AST in a urinary tract infection [146] and to study the non-lethal effects of Ag nanoparticles on a gut bacterium [147] by monitoring bacterial growth. It is reasonable to believe that this method will be useful for determining the antimicrobial activity of ENMs including via portable mass spectrometry [148].

#### Electronic nose and electronic tongue

The electronic nose and electronic tongue are combinations of gas and chemical sensors and are non-invasive and portable tools to assess volatile compounds. Gas sensor arrays are ‘electronic noses,’ and chemical sensor arrays are ‘electronic tongues’ [144, 149]. Typically, they offer fast response and require little or no sampling operations, making them ideal tools for use as on-line monitoring. The cost of these arrays is relatively lower than chromatography, liquid chromatography and mass spectrometry [150]. Electronic noses and electronic tongues have been broadly applied in determining microbiological properties, even the process of growth in complex matrices [150–152]. Thus, they are promising for determining the antimicrobial activity of ENMs provided that their sensitivity and accuracy are improved [152].

#### Isothermal microcalorimetry

The microbial growth involves metabolic processes, which generate heat. The heat flow rate is proportional to the reaction rate, and the total heat produced per unit time is proportional to the extent of the reaction taking place in time. This makes isothermal microcalorimetry a useful, non-specific tool for assessing the process of microbial growth in real time with the integration of proper mathematical models [64, 153–156]. For instance, Bonkat et al*. *[153] reported an isothermal microcalorimetry method for on-line monitoring of microbial growth that offered continuous data with high temporal resolution. It could detect the metabolic activity of bacteria in complex samples [154] and perform AST of bioactive glass in powder formulations [156].

### Advanced gene analysis-based methods

Despite the difficulty of real time and accurate measurements due to sampling requirements and other complicated upstream operations, gene analysis has a significant advantage in terms of high-throughput screening [136]. Gene analysis-based methods will likely remain one of the primary techniques for studying the antimicrobial activity of ENMs in complex matrices over the next decade, especially due to miniaturization of setups and introduction of advanced amplification techniques.

#### Detection of resistance genes

In general, the mainstream of AST using gene analysis method is attributed to the rapid, direct, sensitive and specific detection of resistance genes [32]. Unlike common AST, the mainstream of assessing the antimicrobial activity of ENMs relies on the quantification of biomass via amplification and determination of 16S rRNA gene [30]. The resistance of microbes to ENMs is increasingly studied [83, 86, 157, 158]. Metch et al*. *[83] reported that the *E. coli* 013, *P. aeruginosa* CCM 3955, and *E. coli* CCM 3954 could develop resistance to Ag nanoparticles after repeated exposures. This resistance evolved only a phenotypic change rather than a genetic change. Ewunkem et al*. *[158] reported the nature of the genomic changes responsible for the resistance of bacteria to Fe nanoparticles. Prior knowledge of specific resistance genes is present, and thus it will be a promising approach to assess the antimicrobial activity of ENMs by determining special resistance genes.

#### Rapid and point-of-care gene analysis

Recently, a few isothermal DNA/RNA amplification methods have been developed including loop-mediated isothermal amplification (LAMP), recombinase polymerase amplification (RPA), helicase-dependent amplification (HDA), cross-priming amplification (CPA), nucleic acid sequence-based amplification (NASBA), single primer isothermal amplification (SPIA), rolling circle amplification (RCA) and strand exchange amplification (SEA) [67, 159]. Contrary to PCR and sequencing techniques, isothermal techniques are rapid and can be completed on-site for detection in low-resource settings without much processing of samples [159, 160]. For assessing the antimicrobial activity of ENMs in complex matrices, they will go beyond PCR-based methods becomes of the attractive efficiency, affordability and user-friendliness.

#### eDNA technique

eDNA is the DNA directly extracted from environmental samples, including soil, sediment, water or air, without enrichment, which is now being used to detect individual species and communities in ecosystems [96]. eDNA technologies provides a full spectrum for assessing adverse effects by environmental stressors including that from pollutants at different levels of biological organization. They are a key advanced tool for evaluating the effects of pollutants on wildlife with time/labor savings and non-invasive sampling [161, 162].

### Integrated comprehensive droplet digital detection

**Fig. 7:**
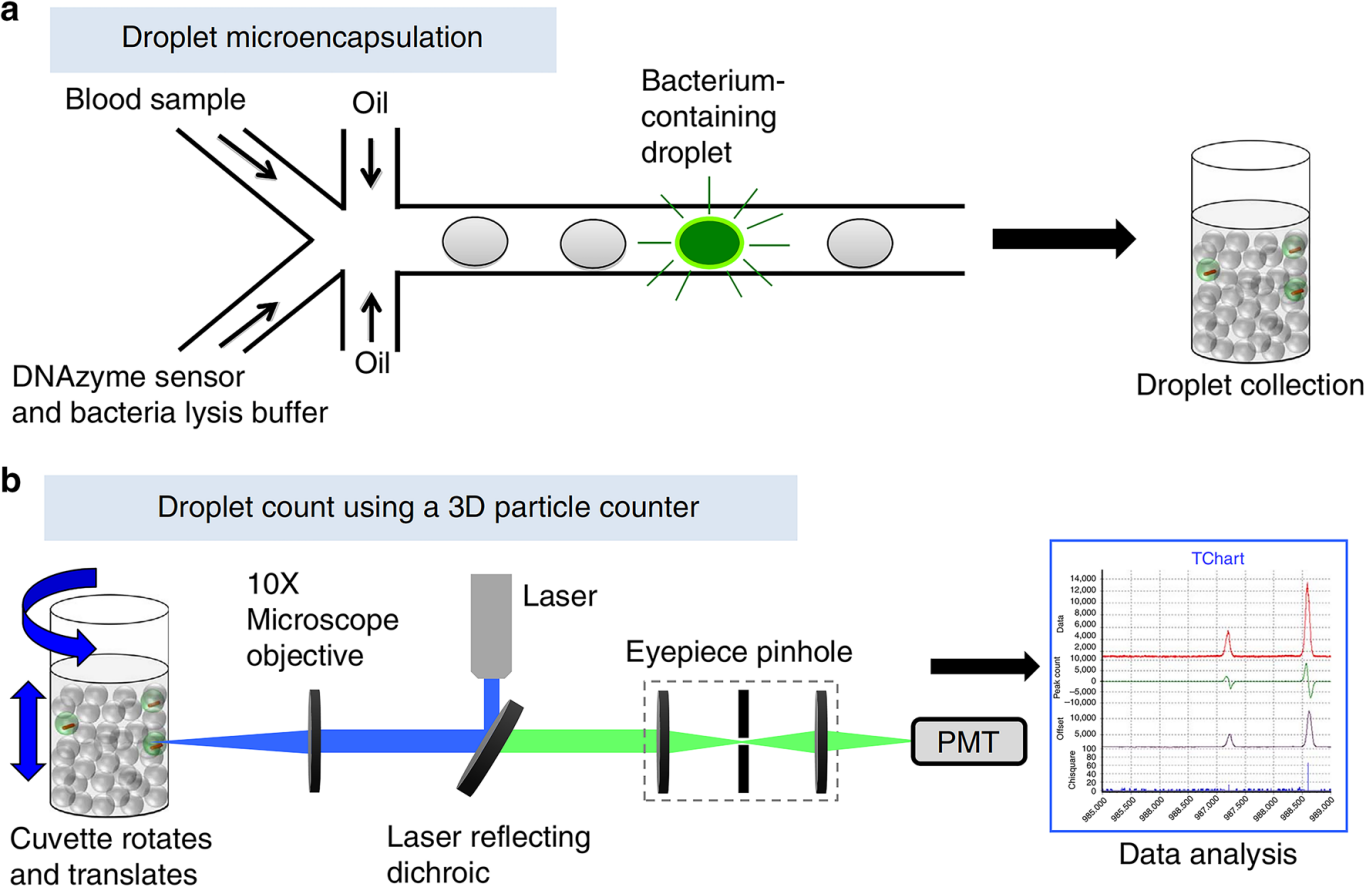
Schematic of integrated comprehensive droplet digital detection [163]. **a** Blood samples and DNAzyme sensors are mixed and then encapsulated in droplets. DNAzyme sensors produce an instantaneous signal in the droplets that contain the bacterium. **b** Droplets are collected and analyzed using a high-throughput 3D particle counter that permits accurate detection of single-fluorescent droplets in a milliliter pool of non-fluorescent droplets within minutes

Kang et al*. *[163] developed a technology called integrated comprehensive droplet digital detection to rapidly (1–3 h) and selectively detect bacteria directly from a large volume of unprocessed blood in a one-step, culture-free reaction. Their technology integrated real-time, bacterium-detecting fluorescence chemistries, droplet microfluidics and a high throughput particle counter system (Fig. 7). Using *E. coli* as a target, the method could selectively detect both stock isolates of *E. coli* and clinical isolates in spiked whole blood at single-cell sensitivity. This provides absolute quantification of target bacteria within a broad range of low concentrations with LODs in the single-digit regime [67]. This method may be used to rapidly measure the results of ENM-microbe interactions.

### Combined methods

There is no single gold standard for characterizing the interaction of ENMs with microbiota. A combination of different analytical methods is highly recommended as a strategy to compensate for the drawbacks and limitations of individual methods [2]. In some previous studies, more than two methods were frequently used to characterize the effects of ENMs on microbes in complex matrices [22, 36, 51, 81, 87, 88, 104, 111, 164, 165]. For instance, Samarajeewa et al*. *[81, 111, 165] combined plate counting, respiration, protein analysis, PCR-DGGE and DNA-sequencing methods to study the effects of ENMs on microbial community in soil. Miao et al*. *[87, 88, 164] combined confocal laser scanning microscopy, OD, high throughput screening and DNA-sequencing methods to study the effects of ENMs on microbes in complex matrices (e.g., sediment). The multi-aspect characterizations as a consequence of the combination of analytical methods will improve reliability and comprehensiveness.

## Conclusion

**Fig. 8:**
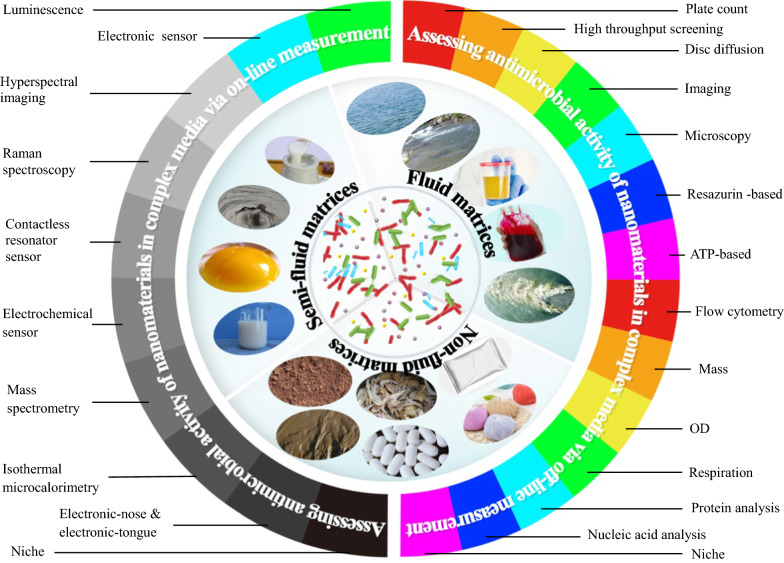
Applied and emerging analytical methods for the assessment of antimicrobial activity of ENMs in complex liquids, semi-liquids, and non-liquid matrices. Applied methods are marked with bright colors. Emerging methods are indicated by pale colors

The full scale and scope of the antimicrobial activity of ENMs in complex matrices are of great significance for both fundamental research and applications. Several methods have been developed to address this task via on-line and off-line measurements. Other promising methods are emerging, thus enabling better characterization in liquid, semi-liquid and non-liquid matrices (Fig. 8).

**Fig. 9:**
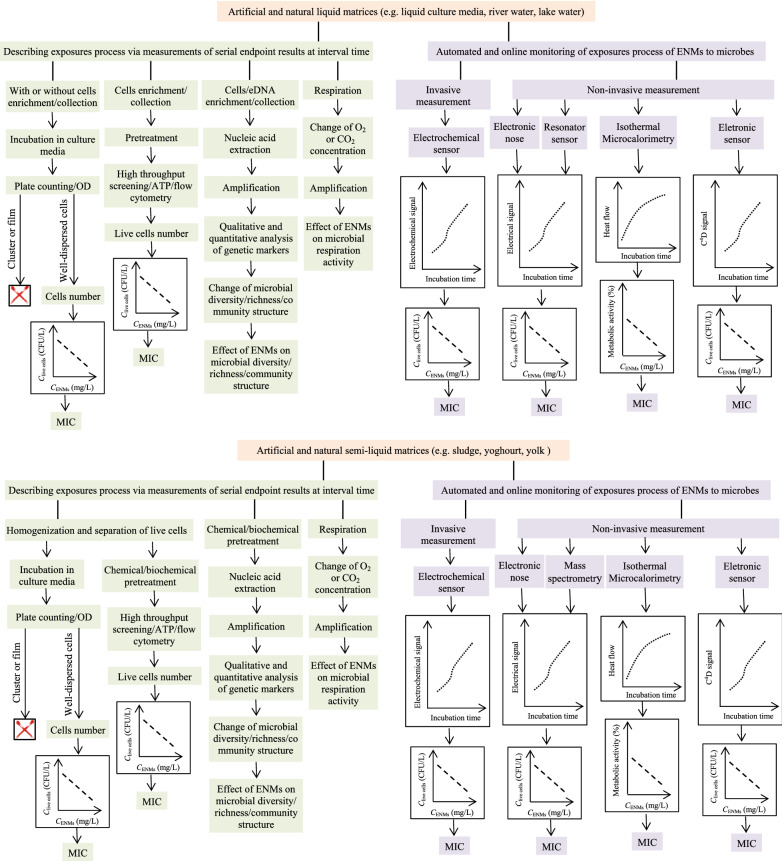

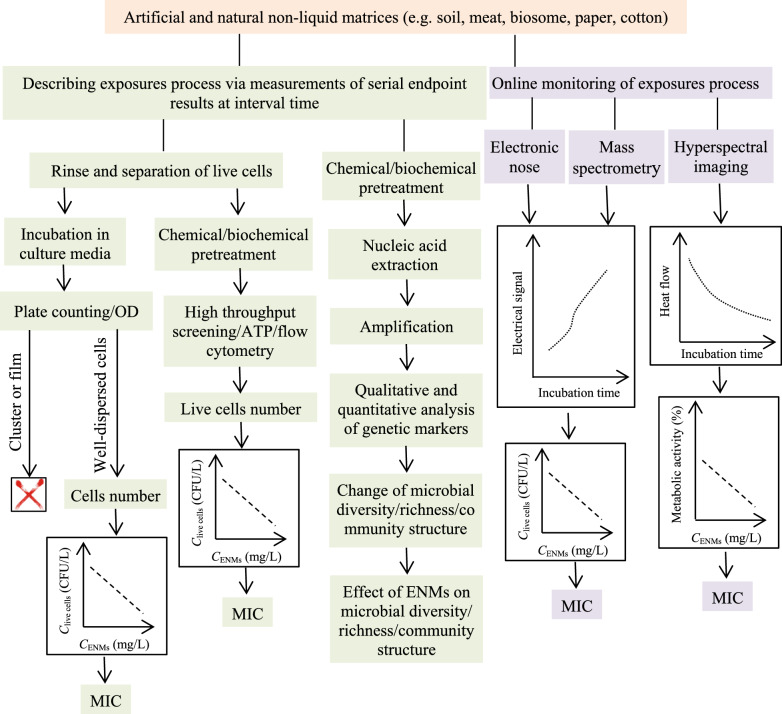
Recommended strategies for assessing antimicrobial activity of ENMs in complex liquids, semi-liquids and non-liquid matrices

Ideally, researchers need a method or a few techniques to present reliable and comprehensive results including accurate MIC values. Considering cost, user-friendliness, time consumption, sensitivity, accuracy and stability, we provide some general recommendations on the use of these present methods with the most feasibility to yield appropriate data on ENM-microbe response (Fig. 9). Our goal was to provide the community with current information on the most appropriate strategies depending on the particular needs and resources for their experimental setup. Of course, researchers should systematically investigate the performance of their application before the establishment of standard/reference methods with new techniques.

To assess the antimicrobial activity of ENMs, common analytical methods were studied in comparison [15, 31, 100, 119]. These counterpart methods presented remarkably different results for the same sample. To date, no reports have compared results among different laboratories, likely because most studies mainly focused on scientific advancements rather than practical applications. The lack of standardized guidance on the analytical methods exacerbates the uncertainty in antimicrobial activity data interpretation [11, 113]. Therefore, international standardization is urgently needed in the fields of ecological environment, food, pharmaceutics and materials science.

## Data Availability

Not applicable.
